# Eight Good Reasons for Careful Monitoring and Evaluation of the Vaccine Campaign against COVID-19: Lessons Learned through the Lombardy Experience for Dealing with Next Challenges

**DOI:** 10.3390/ijerph19031073

**Published:** 2022-01-19

**Authors:** Giovanni Corrao, Guido Bertolaso, Giovanni Pavesi, Letizia Moratti

**Affiliations:** 1 National Centre for Healthcare Research and Pharmacoepidemiology, University of Milano-Bicocca, 20126 Milan, Italy; 2 Unit of Biostatistics, Epidemiology and Public Health, Department of Statistics and Quantitative Methods, University of Milano-Bicocca, 20126 Milan, Italy; 3 Vaccination Campaign Management, Lombardy Region, 20124 Milan, Italy; bertolaso1@gmail.com; 4 General Directorate of Welfare Department, Lombardy Region, 20124 Milan, Italy; giovanni_pavesi@regione.lombardia.it; 5 Welfare Councillor, Lombardy Region, 20124 Milan, Italy; letizia_moratti@regione.lombardia.it

**Keywords:** effectiveness, impact, natural experiment, observational studies, protection persistence, risk factors, safety, vaccine campaign, vaccine platform, variant of concern, vulnerability

## Abstract

Background: Using the knowledge gained during the first eleven months of the vaccine campaign in Lombardy, Italy, we provide an overview of the benefits of using reliable, complete, and rapidly available observational data to monitor the progress of the vaccine strategy. Methods: A population-based platform was implemented by linking four registries reporting individual data on: (i) date, type, and dose of vaccine dispensed; (ii) SARS-CoV-2 infections and hospital admissions and deaths due to COVID-19; (iii) inpatient diagnoses and outpatient services supplied by the Regional Health Services (RHS); and the (iv) health registry reporting and updating data on patient status. Background, methods, findings, and implications of eight COVID-19 relevant questions are reported. Results: Before starting the vaccine campaign, we identified high-risk individuals who need to be prioritized. During the vaccine campaign, we: (i) monitored the trend in the speed of the vaccine campaign progression and the number of prevented clinical outcomes; (ii) verified that available vaccines work in real-life, assessed their effectiveness-harm profile, and measured their reduced effectiveness against the delta variant. Finally, we studied the reduced effectiveness of the vaccine over time and identified risk factors of post-vaccine infection and severe illness. Conclusions: The correct use of rapidly available observational data of good quality and completeness generates reliable evidence to promptly inform patients and policymakers.

## 1. The Ongoing Vaccine Campaign Is a Remarkable ‘Natural’ Experiment

Data for international comparisons of vaccination rates aimed at identifying critical issues and ensuring global efforts against the pandemic are now readily available from several worldwide organization databases. Although providing an overall overview of the vaccination campaign across the world, ecological comparisons do not address all issues, for example, (i) who should receive the vaccine early (to tackle priorities [1]), (ii) who has received the vaccine (to reduce health inequality [2]), (iii) the impact of the vaccine campaign on preventing SARS-CoV-2 infections and COVID-19 disease and death (and how it changes according to the emerging variants of concern [3]), or (iv) issues on the safety of vaccines (beyond classical pharmacovigilance methods [4]).

With the knowledge gained in the first eleven months of the vaccine campaign in Lombardy, the most populous region of Italy, this paper provides an overview of methods to improve the ongoing vaccine campaign by using available observational data.

## 2. Developing a Vaccine Integrated Platform

**Figure 1:**
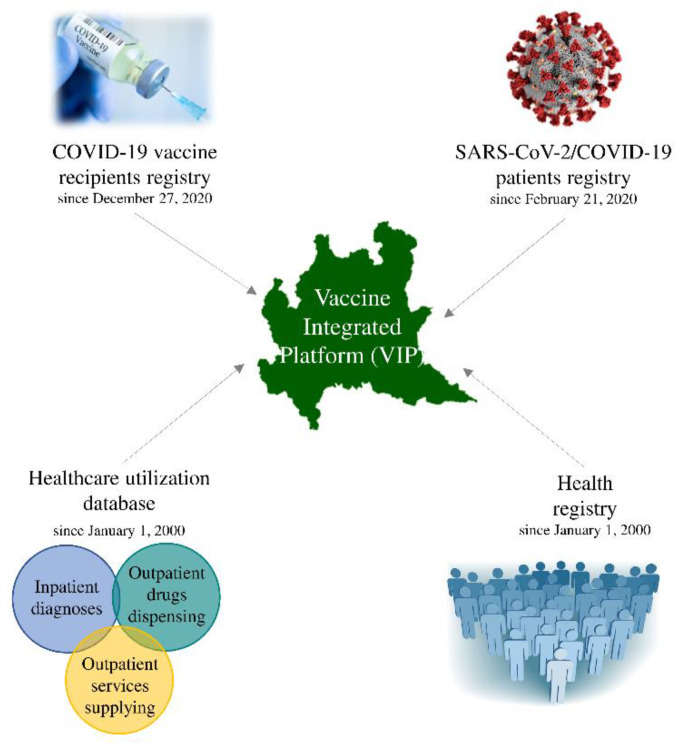
Data sources for developing the Lombardy Vaccine Integrated Platform.

As shown in Figure 1, four data sources were used to develop the Lombardy Vaccine Integrated Platform (LVIP). First, at the start of the vaccine campaign (27 December 2020), a vaccine registry was promptly established by the Regional Health Authority to collect individual data on the date, type, and dose of vaccine dispensed. This first source will be hereafter referred to as the COVID-19 Vaccine Recipients registry. Second, the registry of patients with a confirmed diagnosis of SARS-CoV-2 infection was established on the 21st of February 2020 (i.e., the date of the first confirmed diagnosis in Lombardy) to monitor SARS-CoV-2 infections and hospital admissions, emergency room access, and deaths due to COVID-19. This second source is hereafter referred to as the SARS-CoV-2/COVID-19 patient registry. Third, the healthcare utilization database that since the year 2000 collects various types of data, including inpatient diagnoses supplied by public or private hospitals, and outpatient drug and services supplied by the RHS departments, was also used to collect data on the health profile of the population. This third source is hereafter referred to as the Healthcare Utilization Registry. Finally, the health registry reports and updates data on patients’ status of the Lombardy RHS, which reports date and causes of entry (birth, immigration) and exit from (death, emigration) the database of resident in the region. These different types of data can be interconnected since all databases use a unique individual identification code. To maintain privacy, each identification code is automatically made anonymous, and the individual can only be identified by the Regional Health Authority on request from judicial authorities.

The LVIP design was based on the following features. The first aim was building a (dynamic) population on which the platform is based, and so potential vaccine recipients were selected. This implies that the platform has a population-based structure (i.e., individuals who received the vaccine in Lombardy, although residents outside the region were excluded) and the corresponding data were collected at the patient level (suggesting that an approach meeting good clinical practices should be therefore adopted) [5]. Our second aim was to characterize each vaccine recipient with relevant features at baseline. We associated the COVID-19 Vaccine Recipients registry records with Healthcare Utilization (diseases/conditions of candidates) and SARS-CoV-2/COVID-19 patient (prior SARS-CoV-2 infection/COVID-19 disease) registries. Third, the platform is continuously updated through record linkage with COVID-19 Vaccine Recipients (vaccine exposure), SARS-CoV-2/COVID-19 patient (events that vaccine should prevent) and Healthcare Utilization (other events) registries. Finally, a very large population of vaccine recipients was included. Potential vaccine recipients were 9.1 million citizens on the 27 December 2020 and 7.6 million of them received at least one dose on the 9th of November 2021. This allowed us to obtain very stable estimates, even when very rare events were investigated.

## 3. Generating Evidence from the Vaccine Integrated Platform

**Figure 2:**
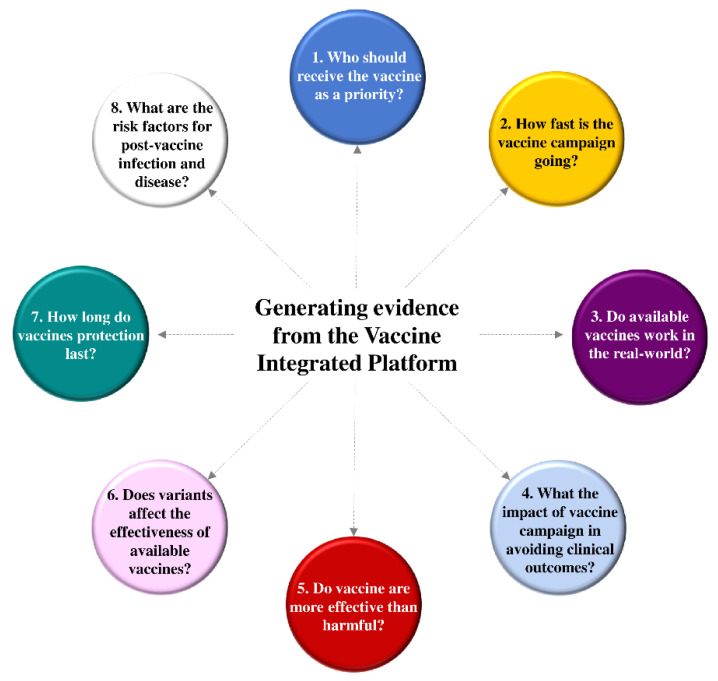
Eight research questions which have been answered for improving managing and evaluating the ongoing vaccine campaign.

As LVIP was designed for answering several relevant questions, it can be considered a research infrastructure suitable for managing the vaccine campaign. Nonetheless, data availability does not ensure obtaining credible evidence [6,7,8]. Study design and data processing must be considered as a bridge between the research question to be answered and the implications of available evidence to make clinical decisions. Herein, we investigated eight vaccination-related questions and discuss the research findings (Figure 2). The Strengthening the Reporting of Observational Studies in Epidemiology (STROBE) statement guided all the performed studies.

**Figure 3:**
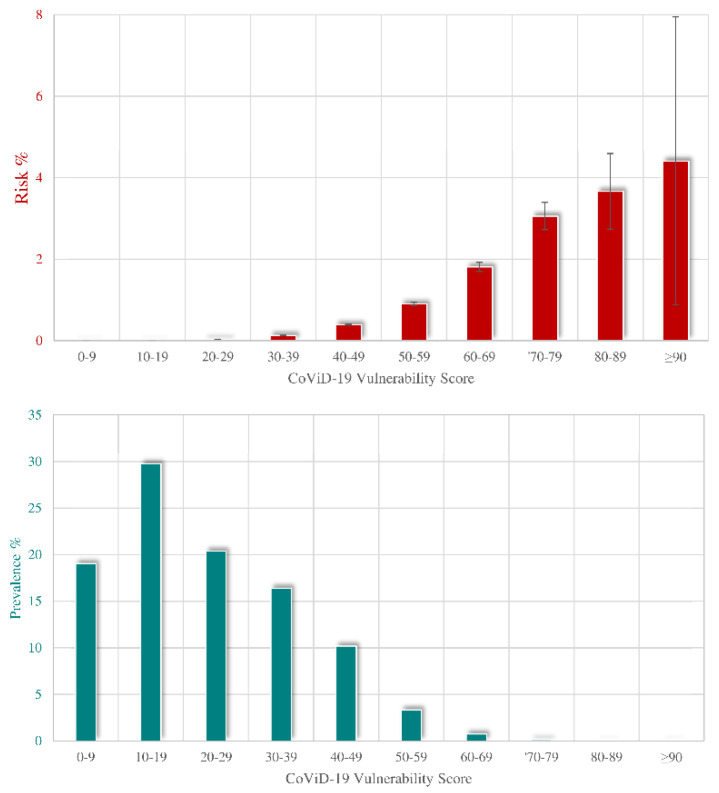
Relationship between categories of COVID-19 Vulnerability Score (CVS) and (i) the risk of occurrence of severe/fatal forms of COVID-19, with 95% confidence band (upper box, red columns), (ii) its distribution among NHS beneficiaries (bottom box, green columns). Columns indicate the observed values of risk and prevalence respectively (please see text, Question 1). Footnote. The analysis was based on the cohort of 7,655,502 beneficiaries of the Lombardy Region Health Service for at least two years, who on 21st February 2020 were alive, were aged 18 to 79 years, and did not reside in a nursing home. During the first epidemic wave (until June 2020), this cohort experienced 9160 severe (intensive care unit admitted and mechanically ventilated via intubation) and/or fatal outcomes. The average incidence rate during the first wave was therefore 12.0 cases per 10,000 people at risk.

First question: “Who should receive priority vaccination?” Background. At the beginning of the campaign, the vaccine demand exceeded the available supply, raising a debate regarding the criteria for establishing who should have access to early vaccination [9]. Based on ethical reasons, rationing approaches gave priority to high-risk groups. We aimed to develop and validate a novel prognostic score (which we called the COVID-19 vulnerability score or CVS) to predict critical/severe clinical manifestations of SARS-CoV-2 infection [10]. Methods. The study included 7,655,502 beneficiaries of the Lombardy RHS who on 21st February 2020 were aged 18 to 79 years (training cohort). Overall, 9160 of them experienced severe and/or fatal COVID-19 outcomes during the first wave of the pandemic (until June 2020). Each member of the training cohort was grouped according to 61 conditions/diseases which we identified as candidate predictors of the disease outcome. Multivariate logistic regression was fitted to identify clinical characteristics associated with the outcome of interest. A weight was assigned to each selected parameter by using the coefficient estimated from the model. Weights were sequentially summed to produce the CVS score. The CVS performance was validated by applying the model to several validation sets concerning (i) the second wave that occurred in Lombardy, and (ii) the pandemic period between March to December 2020 that occurred in four regions located in Northern (Valle d’Aosta), Central (Marche), Southern (Puglia), and the islands of (Sicily) Italy. This validation cohort included 15.4 million individuals and 7031 outcomes. Results. Sex, age, and 29 medical conditions that independently predicted the outcome were selected. Supplementary Table S1 reports the list of the selected conditions jointly with the corresponding weights. The areas under the receiver operating characteristic (ROC) curve ranged from 0.85 to 0.88 without significant difference in the discriminant power between periods and regions. A remarkable calibration of observed and predicted outcome probability was observed. The probability of experiencing the outcome of interest was positively associated with CVS, the risk being lower than 0.05% for CVS value ≤ 29, progressing to 2% for a CVS value between 60 and 69, and reaching a much higher value (around 4%) for CVS values ≥ 80 (Figure 3, upper box). Sixty-nine percent of NHS patients had a CVS value ≤ 29, almost 30% ranged from 30 and 69, and less than 1% (0.16%) exhibited a CVS value ≥ 70 (Figure 3, lower box). Implications: A score based on data used for public health management was used to identify priorities for vaccination [11].

Second question: “What is the speed of the ongoing vaccine campaign?” Using the database allowed for the real-time tracking of the number of vaccine recipients that have received the first, second and booster doses. Our data show that to date (15 December 2021), 8,134,947 individuals (89% of recipients) have received at least one dose, almost all of them (7,906,437) have received two doses of vaccine manufactured by Pfizer, Moderna, Oxford–AstraZeneca, or one dose of the vaccine manufactured by Janssen, of whom 2,275,957 also received the booster. This goal was reached during the first 12 months of the campaign and this corresponds to the administration of 5890 doses per day.

Third question: “Do the COVID-19 vaccines work in the real world?” Background. Although the efficacy of authorized vaccines has been widely demonstrated by randomized clinical trials (RCTs) [12,13,14,15,16,17], the exclusion of more frailty citizens from RCTs limit their generalizability. We assessed the effectiveness of the vaccine as soon as enough individuals received the first dose. Methods. Among potential vaccine recipients, those who received the first vaccine dose were identified. When an individual was vaccinated on a given day (i.e., the index date), a control was randomly selected from the remaining vaccine recipients of the same age and sex. Observational data were collected starting from 14 days after the index date [18] and were terminated at the first sign of COVID-19, (SARS-CoV-2 infection; hospital admission for COVID-19; severe COVID-19 reported during admission to an intensive care unit or death), emigration, death unrelated to COVID-19, or the end of the study period (observation ended on 31 May 2021). When an unvaccinated control was vaccinated, follow-up was halted on that date for both the unvaccinated control and the corresponding vaccinated cohort member. Person-months were partitioned into subperiods categorized as unexposed, exposed to the first dose, and exposed to both doses of vaccine. A Cox proportional hazard model was used for estimating the hazard ratio (HR) of the considered outcomes, together with a 95% confidence interval (CI), associated with the time-dependent exposure to the vaccine. Vaccine effectiveness was measured as complementary to the HR. The model was adjusted for available demographic and clinical characteristics. Results. In total, 2,351,853 individuals were included in this study as they had received at least one dose of vaccine. Partial and complete vaccination reduced the risk of infection by 84% and 90%, hospitalization by 90% and 97%, and intensive care admission and mortality by 93% and 99%, respectively (Table 1). Implications. Although the campaign involved individuals who were excluded from RCTs, the effectiveness of vaccines was consistently confirmed based on real-world experience [19].

**Figure 4:**
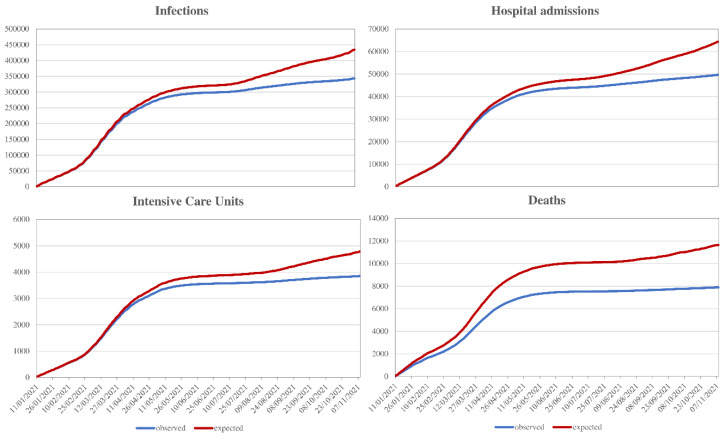
Trend in the cumulative number of infections, hospitalizations, accesses in intensive care units and deaths observed and expected (i.e., that would have occurred in the absence of the vaccination campaign) during the first eleven months from starting the campaign (please see text, Question 4). Footnote. The analysis was based on the cohort of 9,140,390 citizens from Lombardy, beneficiaries of the RHS, who, having already turned ≥12 years old on 27 December 2020, or celebrating their 12th birthday by 11th November 2021, were to be considered potential vaccine recipients for the current study. Estimates were based on the so-called prevented fraction (PF_d_ = P_d_(1-HR)) where P_d_ is the cumulative proportion of citizens reached by the vaccine up to day (d) (see answer to the question 2) and HR is the risk ratio measuring the association between exposure status and a given outcome (see answer to the question 3). The HR is thought to be invariable over time (although this assumption may be questioned; see the answer to the question 6). In contrast, P_d_ increased during the campaign. The number of outcomes avoided from campaign starting until the day (d) was calculated by applying to the number of outcomes occurred up to day (d) the PF value calculated up to 14 days earlier. The number of outcomes that would have been observed if the vaccine campaign had not been implemented derived from the outcomes avoided and those observed.

Fourth question. “What is the impact of the vaccine campaign in avoiding clinical outcomes?” Background. Under the same effectiveness, the vaccine campaign may have a different impact on reducing outcomes according to the speed with which the campaign proceeds, but also the characteristics of the individuals to whom the vaccine is administered. The impact of intervention in preventing selected clinical outcomes according to vaccine campaign progression was estimated. Methods. The impact was measured through the so-called preventable fraction [20]. The number of outcomes avoided from the starting the campaign until at a given day (d) was modeled as a function of the number of outcomes occurred until (d) and the hazard ratio for the association between vaccination status and the outcome risk (please see answer to question 3). The number of outcomes that would have been observed if the vaccine campaign had not been implemented derived from the outcomes avoided and those observed. Results. In the period between 27 December 2020 to 9 November 2021, vaccine administration prevented 3579 deaths (11,473 would have occurred in the absence of a vaccine, 7894 were observed during the campaign), 933 admissions to the intensive care unit (4785 vs. 3852), 14,713 hospitalizations (64,348 vs. 49,635), and 90,875 infections (434,033 vs. 343,158) in the target population (Figure 4). Implications. These data represent the clearest evidence of what would have happened if the vaccination campaign had not been initiated and conducted according to priorities.

**Figure 5:**
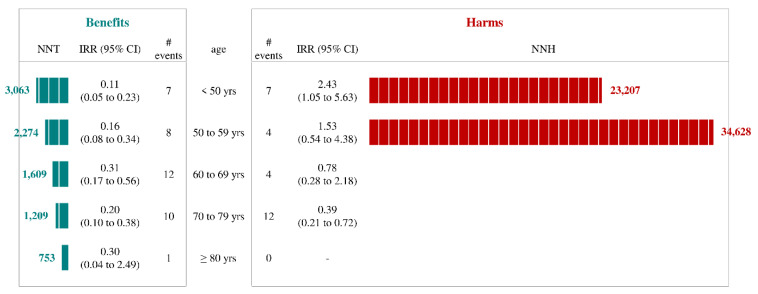
Comparing benefits (number needed to treat) and harms (number needed to harm) of Oxford–AstraZeneca vaccine administration to women according to age category (please see text, Question 5). Footnote. The analysis was based on the cohort of 755,557 citizens who from 30 January to 3 May 2021 received the first dose of Oxford–AstraZeneca vaccine at the index date, and on as many 1:1 matched recipients who on the index date had not yet received any vaccine dose. Study outcomes included events which are expected to be avoided by vaccination (i.e., hospitalization and death from COVID-19) and those which might be increased after vaccine inoculation (i.e., venous thromboembolism). Incidence rate ratios (IRR) of vaccinated and unvaccinated citizens were separately estimated within strata of gender and age category. When suitable, number needed to treat (NNT) and number needed to harm (NNH) were calculated to evaluate the balance between benefit and harm of vaccines within each gender and age category.

Fifth question: “Are vaccines more effective than harmful?” Background. Concerns about the safety of vaccines have been spreading since mid-March 2021 [21] when the Oxford–AstraZeneca vaccine was halted in several European countries due to reports of thromboembolic events [22,23,24]. We compared the benefits and safety of the different vaccines in subpopulations defined by age and sex. Methods. The protocol used for assessing effectiveness was integrated with a similar protocol aimed at assessing safety (please see the Methods section for addressing the third question). This new study was designed by randomly selecting 10 controls from potential vaccine recipients who did not have yet received the vaccine once the vaccination of an index cohort member occurred. Follow-up started at the index date and was halted to the date of outcome occurrence, or censoring (emigration, death from any cause, or 28 days after the index date). Follow-up was interrupted for all the members of a given 1:10 risk-set, at the earliest date when an unvaccinated control was vaccinated. Different metrics were used to compare subperiod incidence rates, i.e., calculating the number needed to treat (NNT) and to harm (NNH) measured vaccine benefits and vaccine safety, respectively. Results. By limiting the observation to the sex/age categories with the highest expected risk (i.e., women under 50 years of age), an NNT value of 3063 was observed among individuals who received the Oxford–AstraZeneca vaccine, the corresponding NNH value being 23,207. A stronger unbalanced risk–benefit profile was observed among women aged 50–59 years with 2274 NNT and 34,628 NNH values (Figure 5). There was no evidence that the vaccine increased the risk of venous thromboembolism among women 60 years or older. Implications. A favorable balance between the benefit/risk ratio was observed in the current study, even among younger women who received the Oxford–AstraZeneca vaccine. In this setting, vaccination of a little more than 3000 subjects yielded one unit of therapeutic benefit, while vaccination of >23,000 individuals was required to produce an adverse event.

Sixth question: “Do variants affect the effectiveness of available vaccines?” Background. Variants of concern (VoC) are new variants of SARS-CoV-2 characterized by enhanced transmission, increased pathogenicity, and reduced efficacy of prophylactic and therapeutic measures [25,26]. The Delta variant (DV) has been described as having a viral load ~1000-times higher than the original SARS-CoV-2 [27], making it the predominant variant in Western countries, including Italy [28]. Concerns about the possible reduction in the effectiveness of available vaccines against DV have been raised [29,30]. We compared the protective action of the vaccine against the onset of infection by delta and alfa variants, which account for ~90% of infections occurring in Lombardy in June 2021. Methods. From 27 December 2020 to 16 July 2021, over two million COVID-19 vaccine candidates were tested with a nasopharyngeal swab. Among these, 419,400 individuals (19%) were confirmed COVID-19 positive by polymerase chain reaction (PCR). Whole-genome sequencing was used to identify VoCs in 11,300 individuals. In general, 1279 and 8897 sequenced samples were positive for the Delta and Alpha variants, respectively. A 1:1:10 matching procedure was carried out by selecting all individuals confirmed positive for the Delta variant on a certain date (index date) and matching each Delta case to one individual positive to the alfa variant on the index date and 10 individuals negative up to the index date. Conditional logistic regression models were fitted to estimate the odds ratio and the corresponding 95% CI of SARS-CoV-2 infection caused by a variant associated with natural (previous infection) and induced (partial or complete vaccination) exposure to SARS-CoV-2. Results. Significant protection towards reinfection was observed among individuals with a previous infection, with no between-variant significant differences. Partial and complete vaccination were associated with 29% (95% CI, 7% to 45%) and 75% (66% to 82%) reduced risk infection with the Delta variant, compared to 62% (48% to 71%) and 90% (85% to 94%) risk of infection with the alpha variant (Table 2). Implications. Lower protection against infections caused by the Delta variant was observed compared to the risk of infection from the alpha variant, even after receiving both doses. These findings support efforts to promote complete vaccination and the implementation of individual protection measures, especially because the DV is currently the major variant of concern worldwide.

**Figure 6:**
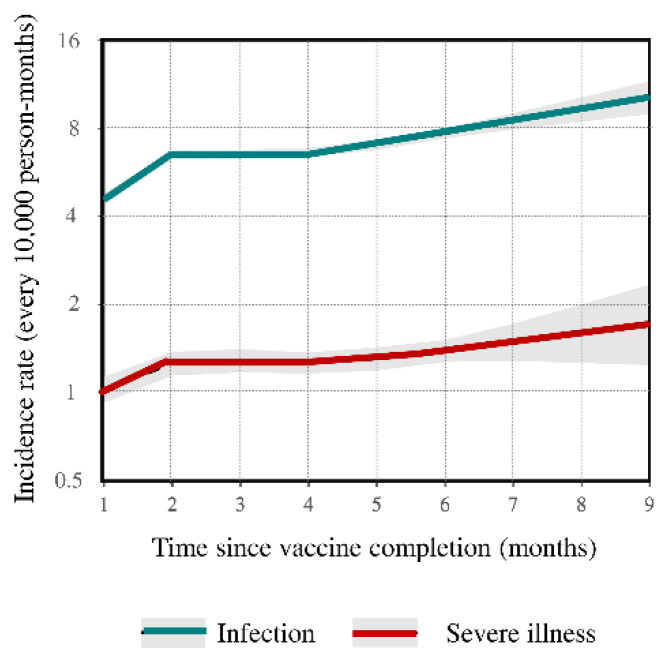
Influence of time since complete vaccination on rates of SARS-CoV-2 infection, green line, and severe COVID-19 illness, red line (please see text, Question 7). Footnote. Estimates based on the cohort of 5,351,085 citizens who received complete vaccination from January to July 2021. The figure reports the trends in age–period–cohort modeled incidence rates (and 95% confidence bands) according to time since complete vaccination. Estimates are adjusted for the month of vaccine completion (“cohort effect”), and the month of outcome occurrence (“period effect”).

Seventh question: “How long does vaccine protection last?” Background. Few studies have investigated the persistence of vaccine-dependent protection by direct approaches (clinical outcomes assessment [12,16,31,32]), rather than surrogate-based (neutralizing antibody assay [17,33,34]) approaches. We evaluated the effects of the time since receiving complete vaccination on incidence rates of post-vaccination SARS-CoV-2 infection and severe COVID-19 illness up to 9 months since vaccine completion [35]. Methods. The 5,351,085 individuals who received the scheduled complete vaccination from 17 January to 31 July 2021 were followed from 14 days after vaccine completion until 20 October 2021. Changes over time in the rate of SARS-CoV-2 infection and COVID-19 hospital admissions or deaths (severe illness) were analyzed. The primary factor of interest was the number of months between complete vaccination and outcome occurrence. To separate the effect of time since vaccine completion from other temporal factors, we adopted the age–period–cohort (APC) model to the setting of interest [36]. Results. In total, 14,140 infections and 2450 cases of severe illness were reported, corresponding to incidence rates of 6.7 and 1.2 cases per 10,000 person-months, respectively. From the first to the nine months since vaccine completion, infection and severe illness rates respectively increased from 4.6 to 10.2 and from 1.0 to 1.7 cases every 10,000 person-months (Figure 6). The increasing infection rate was greater for individuals aged ≥ 60 years who received adenovirus-vectored vaccines (from 4.0 to 23.5 cases every 10,000 person-months). Implications. Although the risk of post-vaccination infection and severe illness remains low, the fast waning of vaccine-induced immunity, particularly for individuals who received the adenovirus vaccine, suggests that the campaign of the third booster dose should be accelerated.

Eighth question: “What are the risk factors for post-vaccination infection and disease?” Background. With the aim of understanding risk factors for post-vaccination SARS-CoV-2 infection and its clinical manifestations [37], we explored selected characteristics associated with an increased risk of post-vaccination SARS-CoV-2 infection and severe COVID-19 illness [38]. Methods. A cohort of 5,351,085 individuals aged 12 years or older who received a complete vaccination from 27 December 2020 until 31 July 2021 were followed from 14 days after vaccine completion until 11th November 2021. During follow-up, 17,996 infection cases and 3023 severe illnesses cases occurred. For each case, controls were randomly selected to be 1:1 (infection) or 1:10 (severe illness) matched for date of vaccination completion and municipality of residence. The association between selected candidate predictors (sex, age, total number of previous contacts with RHS, previous occurrence of SARS-CoV-2 infection, type of received vaccine, and the presence of 59 comorbidities) and outcomes were assessed through multivariable conditional logistic regression models. Results. Table 3 shows that (i) as age increased, there was a clear trend towards decreasing the odds of infection and increasing odds of severe illness; (ii) male gender was a significant risk factor for severe illness; (iii) as the number of contacts with RHS increases, a trend towards increasing odds of both infection and severe illness was observed; (iv) previous history of SARS-CoV-2 infection and mRNA-based vaccination were significant protective factors against infection and severe illness. Significantly higher odds of infection and severe illness were respectively associated with 14 and 34 comorbidities. Implications. An extensive set of factors was identified that were associated with an increased risk of post-vaccination SARS-CoV-2 infection and/or severe COVID-19 illness. Our findings suggest that post-vaccination vulnerability to severe clinical manifestations of SARS-CoV-2 infection may be mainly affected by a poor immune response, possibly due to comorbidities, rather than to other specific disorders.

## 4. Summarizing Evidence and Expanding the Platform Use

The prompt implementation of research infrastructure as the LVIP, and the appropriate study design and data processing for generating real-world evidence allowed for careful monitoring of the effectiveness of COVID-19 vaccines to provide scientific evidence to policymakers. Before starting vaccination, we identified high-risk individuals who were prioritized (please see question 2). During the vaccine campaign, we (i) carefully monitored the trend in vaccination rates (question 1) and the number of prevented SARS-CoV-2 infections, and severe/fatal COVID-19 (question 4) as the vaccine campaign progressed; (ii) verified that available vaccines work in real life (question 3); (iii) assessed the benefits/risks (question 5); (iv) effectiveness of the vaccine against variants of concern (question 6). Finally, we studied the vaccine protection against COVID-19 over time to assess the suitability of a campaign for administering a booster (question 7) and for identifying vaccinated individuals at higher risk of infection and severe illness (question 8). Of interest, further studies not directly linked with the vaccine campaign were performed by LVIP. For example, since April 2020, a timely and quick analysis showed that the increased use of medications for lowering the blood pressure in COVID-19 patients reflected the background hypertension and other cardiovascular (CV) disease comorbidities, which are factors associated with an increased risk of infection, rather than the use of ACE inhibitors and angiotensin receptor blockers as initially hypothesized [39]. Yet, methodologic advancements for investigating the changes in healthcare resources during the pandemic have been recently proposed [40].

## 5. Strengths, Pitfalls, Methodologic Challenges

The strengths of our approach have been discussed above in the Section 2. In summary, the main advantages of our approach include the platform design (i.e., the population-based structure), the stored data (i.e., information collected at individual level and the availability of a large set of demographic and clinical data), the target population (i.e., its large size), and the ability to promptly respond to important public health concerns, such as the timely identification of high-risk individuals [10] or the assessment of the safety of using of ACE inhibitors and angiotensin receptor blockers [40].

This study has several potential limitations. All studies were based on secondary real-world data that were collected for managing healthcare databases rather than for research purposes. Several sources of bias should be considered when interpreting the results. Systematic uncertainty could have been generated by misclassification of exposure (e.g., previous infection and/or vaccination), outcome (e.g., positive PCR result of a nasopharyngeal swab), and confounders (e.g., comorbidities). However, while we are confident of the reliability and completeness of vaccination data as systematic controls on official data of vaccine deliveries were carried out, other sources of misclassification are inevitable. For example, the data on previous infections are based on nasal swabs, but we are aware that many infected have tested PCR negative. Furthermore, although only the molecular tests carried out by accredited laboratories have been recorded, sensitivity and specificity ranged between 83% and 96% and between 93% and 96%, respectively [41], and both involve misclassification of the infection status (questions 6 and 8). We aimed to reduce other challenging forms of misclassification. For example, immortal time bias (i.e., a period of the cohort follow-up time during which the outcome of interest cannot occur by design [42]) was systematically considered using time-dependent variables (question 3). The main pitfall in our design is that unmeasured factors may be distributed differently among vaccinated and unvaccinated individuals, thereby confounding the estimated vaccine effectiveness. Basic strategies to reduce the potential for confounding include restriction, matching, stratification, and modeling [43]. However, their use requires the collection of detailed clinical information which is rarely the case for retrospective studies based upon secondary data. Thus, we cannot exclude that our estimates of vaccine effectiveness may be affected by residual confounding factors (question 3, and indirectly to questions 4 and 5). Some extensive thought has been taken to overcome confounders for answering question 7. In our approach, rather than estimating the trend in vaccine effectiveness, we measured the monthly rate of the outcome of interest since the vaccine regimen was completed. If the effectiveness of the vaccine remains stable over time, then the infection and severe illness rate should remain constant. This opens the door for overcoming the above-discussed concern about incomparability of vaccinated and unvaccinated individuals, simply through the inclusion of vaccinated individuals only (a motivating reference on this issue, i.e., on the user-only design, is reported at the end of these comments [44]). Another approach for controlling confounders is the test-negative case–control design [45]. Shortly, comparing vaccination status in people who test positive, such as for a given variant, with those who test negative facilitates the control for factors that are typically difficult to estimate in observational studies, including differences in health-seeking behaviors, access to testing, and case ascertainment (question 6). Finally, in interpreting exploratory investigations aimed to develop a vulnerability score of severe COVID-19 illness (question 1) and to identify risk factors of post-vaccination clinical outcomes expected to be prevented by vaccines (question 8). It should be noted that our search was restricted to comorbidity data collected by administrative databases, i.e., from registries of hospital admissions and dispensed drugs. In addition, health services and treatments supplied by private providers were not included in our analysis.

In summary, the use of the Vaccine Integrated Platform facilitated accelerated training for growing a large team of young researchers for timely studies real-world studies and for tuning novel methods to reliably monitor the progress of the vaccination campaigns. The findings in this study will facilitate the development of novel strategies for COVID-19 vaccination in a real-world setting.

## 6. Future Challenges

Taking advantage of the knowledge gained in this study and that gained during the last few months by studies in other countries [16,18,46], we propose a systematic collaborative data model to be designed, shared, and implemented [47,48]. This model should be capable of (1) international harmonized data collection; (2) carefully addressing the requirements of the European General Data Protection Regulation; (3) allowing the performance of centralized protocol-driven observational studies by means of data extraction from the participating countries, and data processing; and (4) ensuring that reliable data is available to inform patients and policymakers. We suggest the use of this comprehensive approach to improve our understanding of the natural COVID-19 experiment and for mining reliable evidence to best address and implement the available public health resources.

## Figures and Tables

**Table 1:** Effect of partial and complete vaccination on selected outcomes measured according with data accumulated during the first five months since starting the campaign (please see text, Question 3).

	# Events	Vaccine Status	HR	(95% CI)	Vaccine Effectiveness
Positivity to nasopharyngeal swap	291,128	Partial	0.161	(0.159 to 0.164)	84%
		Full	0.100	(0.098 to 0.103)	90%
Admission to ordinary hospital ward	35,736	Partial	0.100	(0.096 to 0.104)	90%
		Full	0.031	(0.029 to 0.033)	97%
Admission to intensive care unit	3450	Partial	0.033	(0.027 to 0.040)	97%
		Full	0.008	(0.005 to 0.013)	99%
Death	6956	Partial	0.073	(0.067 to 0.081)	93%
		Full	0.009	(0.007 to 0.011)	99%

Footnote. The analysis was based on the cohort of 2,351,853 potential vaccine recipients who on 31 May 2021 received at least one dose of vaccine at a given date (index date), and on as many 1:1 matched recipients who on the index date had not yet received any vaccine dose. Cox proportional hazard models were used for separately estimating the hazard ratio (HR) of selected outcomes, together with its 95% confidence interval (CI), associated with the time-dependent partial of full exposure to vaccine. Vaccine effectiveness was measured as complementary to the HR. The model was adjusted for available demographic and clinical characteristics.

**Table 2:** Effects of natural (previous infection) and induced (partial or complete vaccination) exposure to SARS-CoV-2 on the relative risk reduction (RRR) of the onset of infections caused by Delta and Alpha variants, and corresponding 95% confidence interval (please see text, Question 6).

	Controls (N = 4960)	Delta Cases (N = 496)		Alpha Cases (N = 496)		
	N (%)	N (%)	RRR (95% CI) ^†^	N (%)	RRR (95% CI) ^†^	*p*-Value ^‡^
Previous infection						
Unlike	4411 (88.9)	490 (98.8)	0% (reference)	487 (98.2)	0% (reference)	
Ascertained	549 (11.1)	6 (1.2)	90% (76% to 95%)	9 (1.8)	85% (70% to 92%)	0.547
Vaccination						
No	2650 (53.4)	349 (70.4)	0% (reference)	402 (81.1)	0% (reference)	
Partial	876 (17.7)	93 (18.8)	29% (7% to 45%)	65 (13.1)	62% (48% to 71%)	0.001
Complete	1434 (28.9)	54 (10.9)	75% (66% to 82%)	29 (5.8)	90% (85% to 94%)	0.003

Footnote. The analysis was based on the cohort of 496 citizens who from 27 December to 16 June 2021 had infection by the Delta variant. Delta cases were 1:1 matched with citizens affected by Alpha variant and 1:10 matched with persons who had negative molecular test, according to gender, age, and date of molecular ascertainment. ^†^ Relative risk reduction (RRR) calculated as 1- adjusted odds ratio. The latter was estimated with conditional logistic regression, adjusted for the number of previous contacts with the Regional Health Service, use of corticosteroids, drugs for chronic pain, oral anticoagulant agents and insulin, and the presence of anaemias, chronic respiratory disease, dyslipidaemia, depression, hypertension, coronary and peripheral vascular disease, hypothyroidism, epilepsy and recurrent seizures, psychosis, diabetes without insulin therapy, malignancies, other diseases of the respiratory system, other diseases of the digestive system, other diseases of the genitourinary system, gout, autoimmune disease, other diseases of the circulatory system, symptoms, signs and ill-defined conditions, diseases of the skin and subcutaneous tissues, arrhythmia, inflammatory bowel diseases, other mental disorders, heart failure, glaucoma and chronic kidney disease. ^‡^ Chi-square testing the null hypothesis of between-variant homogeneity of the odds ratios.

**Table 3:** Association between age at vaccine completion and other features of the study cohort and the odds of post-vaccine SARS-CoV-2 infection, left panel, and severe COVID-19 illness, right panel (please see text, Question 8).

	Post-Vaccine SARS-CoV-2 Infection				Post-Vaccine Severe COVID-19 Illness			
	Cases	Controls	OR	(95% CI)	Cases	Controls	OR	(95% CI)
Age category								
<40 yrs	3083 (21.2%)	2805 (15.6%)	1.00	(ref.)	84 (2.8%)	2581 (8.5%)	1.00	(ref.)
40 to 59 yrs	7247 (40.3%)	6903 (38.4%)	0.76	(0.70 to 0.83)	384 (12.7%)	6204 (20.5%)	1.60	(1.14 to 2.25)
60 to 79 yrs	3892 (21.7%)	4926 (27.4%)	0.46	(0.42 to 0.51)	660 (21.8%)	6416 (21.2%)	2.48	(1.76 to 3.50)
≥80 yrs	3024 (16.8%)	3332 (18.5%)	0.51	(0.44 to 0.59)	1895 (62.7%)	15,029 (49.7%)	6.99	(4.89 to 9.99)
Sex								
Female	10,023 (55.7%)	10,164 (56.5%)	1.00	(ref.)	1505 (49.8%)	17,299 (57.2%)	1.00	(ref.)
Male	7973 (44.3%)	7832 (43.5%)	1.03	(0.99 to 1.08)	1518 (50.2%)	12,931 (42.8%)	1.41	(1.31 to 1.52)
Contact with RHS								
<5	7430 (41.3%)	7258 (40.6%)	1.00	(ref.)	402 (13.3%)	7674 (25.4%)	1.00	(ref.)
6 to 100	8392 (46.6%)	8815 (49.0%)	1.06	(1.01 to 1.12)	1445 (47.8%)	16,240 (53.7%)	1.60	(1.41 to 1.82)
≥100	2174 (12.1%)	1923 (10.7%)	1.43	(1.31 to 1.56)	1.176 (38.9%)	6316 (20.9%)	3.19	(2.76 to 3.69)
Vaccine type								
mRNA-based	14,432 (80.2%)	14,571 (81.0%)	1.00	(ref.)	2657 (87.9%)	26,617 (88.0%)	1.00	(ref.)
Adenovirus-vectored	3564 (19.8%)	3425 (19.0%)	1.33	(1.24 to 1.44)	366 (12.1%)	3613 (12.0%)	0.97	(0.83 to 1.15)
Previous SARS-CoV-2 infection								
No	17,824 (99.0%)	16,957 (99.0%)	1.00	(ref.)	2922 (96.7%)	28,799 (95.3%)	1.00	(ref.)
Yes	172 (1.0%)	1039 (5.8%)	0.15	(0.13 to 0.88)	101 (3.3%)	1431 (4.7%)	0.67	(0.54 to 0.84)

Footnote. Left panel. Analysis included 17,996 patients who, starting from at least 14 days after completing scheduled vaccine, experienced ascertained SARS-CoV-2 infection documented by nasopharyngeal swab testing positive for the nucleic acids of SARS-CoV-2 (infection cases), and 17,996 controls randomly selected to be 1:1 matched for date of vaccination completion and municipality of residence, and for not having yet experienced the infection on the date on which the corresponding case experienced it (index date). Right panel. Analysis included 3023 patients who, starting from at least 14 days after completing scheduled vaccine, experienced COVID-19 hospital admission, including those in an intensive care unit, or death (severe illness cases), and 30,230 controls randomly selected to be 1:10 matched for date of vaccination completion and municipality of residence, and for not having yet experienced the severe illness on the date on which the corresponding case experienced it (index date). Restricted cubic spline with four knots was used for flexibly modelling the relationship between age and odds of both infection and illness (upper boxes). Adjusted odds ratios, and 95% confidence bands, relative to 40 years old reference age, are presented. Number of cases and controls and corresponding column percentage are reported for each feature considered in the bottom panels. Conditional logistic regression model including all the considered features as covariates were fitted for estimating odds ratios and corresponding 95% confidence interval.

## Data Availability

Data supporting reported results (i.e., the Vaccine Integrated Platform) are kept in the Datawarehouse of the Lombardy Region, under the responsibility of the Regional Health Authority.

## Supplementary Materials

The following are available online at https://www.mdpi.com/article/10.3390/ijerph19031073/s1, Table S1 Prevalence of male gender, age categories and 29 conditions/diseases contributing to the Covid Vulnerability Score (CVS). For each listed contributor, the outcome incidence among the exposed people, the odds ratio (and 90% confidence interval), and the corresponding weight of the contribution to CVS, are reported; Table S2, Diseases and conditions significantly associated with the odds of post-vaccine SARS-CoV-2 infection. The 14 diseases/conditions are sorted for decreasing values of the observed association strength; Table S3, Diseases and conditions significantly associated with the odds of post-vaccine severe COVID-9 illness. The 34 diseases/conditions are sorted for decreasing values of the observed association strength.
