# Supplementary material for: Eight Good Reasons for Careful Monitoring and Evaluation of the Vaccine Campaign against COVID-19: Lessons Learned through the Lombardy Experience for Dealing with Next Challenges
Source: Int J Environ Res Public Health. 2022 Jan 19;19(3):1073. doi: 10.3390/ijerph19031073 (PMC8834613; doi:10.3390/ijerph19031073)
Supplement: Supplementary file 1 [file ijerph-19-01073-s001.zip › ijerph-1510625-supplementary.pdf]

Supplementary material

Table S1. Prevalence of male gender, age categories and 29 conditions/diseases contributing to the Covid Vulnerability Score (CVS). For each listed contributor, the outcome incidence among the exposed people, the odds ratio (and 90% confidence interval), and the corresponding weight of the contribution to CVS, are reported

	No. (%)	No. outcome events	Incidence every 10,000	Odds ratio†	(90% confidence interval†)	Weight‡
Male gender	3,797,636 (49.6%)	6,849	18.0	3.07	2.95 to 3.19	11
Age ≤ 45	3,111,426 (40.6%)	271	0.9	1.00	(reference)	0
Age 46-59	2,305,062 (30.1%)	1,435	6.2	5.95	(5.36 to 6.62)	18
Age 60-69	1,222,310 (16.0%)	2,506	20.5	15.62	(14.09 to 17.32)	27
Age 70-79	1,016,704 (13.3%)	4,948	48.7	27.64	(24.96 to 30.61)	33
HIV infection	31,300 (0.4%)	154	49.2	1.52	(1.33 to 1.74)	4
Other infectious and parasitic diseases	42,422 (0.6%)	443	104.4	1.37	(1.26 to 1.49)	3
Malignancies	177,024 (2.3%)	1,073	60.6	1.42	(1.35 to 1.50)	4
Diabetes without insulin therapy	278,785 (3.6%)	1,419	50.9	1.60	(1.53 to 1.68)	5
Insulin therapy	101,996 (1.3%)	973	95.4	2.35	(2.21 to 2.49)	9
Obesity	16,571 (0.2%)	103	62.2	1.34	(1.13 to 1.58)	3
Disorders of fluid, electrolyte, and acid-base balance	8,576 (0.1%)	135	157.4	1.29	(1.11 to 1.49)	3
Gout	164,428 (2.2%)	1,518	92.3	1.57	(1.50 to 1.66)	5
Coagulation defects	3,603 (0.1%)	36	99.9	1.41	(1.07 to 1.85)	3
Anaemias	613,430 (8.0%)	2,228	36.3	1.51	(1.45 to 1.58)	4
Dementia / Alzheimer	12,671 (0.2%)	145	114.4	1.26	(1.09 to 1.46)	2
Psychosis	138,034 (1.8%)	684	49.6	1.94	(1.80 to 2.08)	7
Depression	588,688 (7.7%)	1,729	29.4	1.35	(1.29 to 1.42)	3
Parkinson’s disease and parkinsonism	40,885 (0.5%)	274	67.0	1.21	(1.09 to 1.34)	2
Epilepsy and recurrent seizures	122,171 (1.6%)	510	41.7	1.37	(1.26 to 1.48)	3
Other diseases of the nervous system and sense organs	35,495 (0.5%)	253	71.3	1.26	(1.13 to 1.40)	2
Ischaemic Heart Disease/Angina	91,539 (1.2%)	845	92.3	1.18	(1.11 to 1.26)	2

Heart failure	21,840 (0.3%)	428	196.0	1.30	(1.18 to 1.43)	3
Vascular diseases	14,936 (0.2%)	217	145.3	1.17	(1.04 to 1.32)	2
Cerebrovascular diseases	35,205 (0.5%)	333	94.6	1.12	(1.02 to 1.23)	1
Hypertension	796,044 (10.4%)	3,136	39.4	1.20	(1.15 to 1.25)	2
Coronary and peripheral vascular disease	658,737 (8.6%)	2,668	40.5	1.75	(1.68 to 1.82)	6
Oral anticoagulant agents	144,713 (1.9%)	1,221	84.4	1.39	(1.32 to 1.47)	3
COPD/Asthma	20,034 (0.3%)	268	133.8	1.15	(1.03 to 1.28)	1
Liver cirrhosis and other liver chronic diseases	29,484 (0.4%)	177	60.0	1.31	(1.16 to 1.49)	3
Chronic kidney disease	17,109 (0.2%)	371	216.8	1.32	(1.20 to 1.46)	3
Diseases of the skin and subcutaneous tissues	106,747 (1.4%)	353	33.1	1.10	(1.00 to 1.20)	1
Chronic pain	191,442 (2.5%)	1,007	52.6	1.28	(1.21 to 1.36)	2
Corticosteroids	935,246 (12.2%)	2,588	27.7	1.62	(1.55 to 1.68)	5
Individuals without any of the 29 conditions above listed	4,600,012 (60.1%)	1,350	2.9	-	-	-

HIV, Human immunodeficiency virus; COPD, Chronic obstructive pulmonary disease

The analysis was based on the cohort of 7,655,502 beneficiaries of the Lombardy Region Health Service for at least two years, who on 21st February 2020 were alive, aged between 18 and 79 years and did not reside in a nursing home. During the first epidemic wave (until June 2020), this cohort experienced 9,160 severe (ICU admitted and mechanically ventilated via intubation) and / or fatal outcomes. The average incidence rate during the first wave was therefore 12.0 cases per 10,000 people at risk

† Odds ratio, and 90% confidence interval, estimated by multivariable logistic regression. Odds ratios measured the strength of the association between the presence/absence of each of the listed contributors and the outcome odds

‡ Weights were obtained from the coefficients of the logistic model; the latter were converted into scores by multiplying them by 10 and rounding them to the nearest whole number

Table S2. Diseases and conditions significantly associated with the odds of post-vaccine SARS-CoV-2 infection. The 14 diseases/conditions are sorted for decreasing values of the observed association strength

	Cases N (%)	Controls N (%)	OR (95% CI)
Chronic kidney disease	170 (0.9%)	100 (0.6%)	1.80 (1.38 to 2.35)
Dementia / Alzheimer	188 (1.0%)	131 (0.7%)	1.62 (1.27 to 2.05)
Transplantation	96 (0.5%)	60 (0.3%)	1.48 (1.05 to 2.08)
Other diseases of the respiratory system	384 (2.1%)	351 (2.0%)	1.40 (1.19 to .64)
Other diseases of the musculoskeletal system	429 (2.4%)	335 (1.9%)	1.38 (1.19 to 1.61)
Heart failure	204 (1.1%)	152 (0.8%)	1.36 (1.09 to 1.89)
Autoimmune haemolytic anaemias, other anaemias, anaemias only tracked from drug therapy	2,004 (11.1%)	1,621 (9.0%)	1.29 1.20 to 1.39)
Gout	764 (4.2%)	642 (3.6%)	1.23 (1.10 to 1.39)
Chronic Obstructive Pulmonary Disease, asthma	1,927 (10.7%)	1,592 (8.8%)	1.23 (1.14 to 1.32)
Arrhythmia	640 (3.6%)	559 (3.1%)	1.20 (1.06 to 1.36)
Chronic pain	707 (3.9%)	610 (3.4%)	1.18 (1.05 to 1.32)
Coronary and peripheral vascular disease	1,612 (9.0%)	1,499 (8.3%)	1.16 (1.08 to 1.26)
Use of corticosteroids	2,310 (12.8%)	2.121 (11.8%)	1.16 (1.08 to 1.24)
Depression	1,943 (10.8%)	1,866 (10.4%)	1.08 (1.00 to 1.16)

Footnote. Analysis included 17,996 patients who, starting from at least 14 days after completing scheduled vaccine, experienced ascertained SARS-CoV-2 infection documented by nasopharyngeal swab testing positive for the nucleic acids of SARS-CoV-2 (infection cases), and 17,966 controls randomly selected to be 1:1 matched for date of vaccination completion and municipality of residence, and for not having yet experienced the infection on the date on which the corresponding case experienced it (index date). Among the 49 conditions tested (i.e., that portion of 59 candidate predictors of whom at least ten cases suffered), the 14 ones significantly associated with the considered outcome are listed in the Table. Adjusted conditional logistic regression models were fitted for estimating adjusted odds ratio, OR (point estimate of the association strength) and the corresponding 95% confidence interval (CI)

Table S3. Diseases and conditions significantly associated with the odds of post-vaccine severe COVID-9 illness. The 34 diseases/conditions are sorted for decreasing values of the observed association strength

	Cases<br>N (%)	Controls<br>N (%)	OR (95% CI)
Chronic kidney disease	113 (3.7%)	289 (1.0%)	2.95 (2.32 to 3.76)
Acute respiratory infections	30 (1.0%)	69 (0.2%)	2.84 (1.80 to 4.47)
Other mental disorders	27 (0.9%)	88 (0.3%)	2.53 (1.59 to 4.04)
Transplantation	33 (1.1%)	156 (0.5%)	2.34 (1.57 to 3.48)
Liver chronic diseases	16 (0.5%)	70 (0.2%)	2.23 (1.26 to 3.93)
Heart failure	173 (5.7%)	488 (1.6%)	2.21 (1.83 to 2.67)
Other diseases of the respiratory system	215 (7.1%)	891 (2.9%)	1.98 (1.68 to 2.34)
Vascular diseases	43 (1.4%)	142 (0.5%)	1.93 (1.35 to 2.76)
Insulin therapy	238 (7.9%)	916 (3.0%)	1.87 (1.59 to 2.19)
Cerebrovascular diseases	112 (3.7%)	453 (1.5%)	1.83 (1.47 to 2.29)
Psychosis	269 (8.9%)	1155 (3.8%)	1.79 (1.54 to 2.08)
Disorders of fluid and acid-base balance	41 (1.4%)	153 (0.5%)	1.71 (1.19 to 2.47)
Dementia / Alzheimer	100 (3.3%)	418 (1.4%)	1.71 (1.36 to 2.16)
Other diseases of the digestive system	183 (6.1%)	877 (2.9%)	1.70 (1.44 to 2.02)
Autoimmune haemolytic anaemias, other anaemias, anaemias only tracked from drug therapy	773 (25.6%)	4121 (13.6%)	1.68 (1.52 to 1.85)
Other diseases of genitourinary system	164 (5.4%	775 (2.6%)	1.67 (1.39 to 2.00)
Symptoms, signs, and ill-defined conditions	115 (3.8%)	534 (1.8%)	1.64 (1.33 to 2.04)
Other disorders of endocrine diseases	37 (1.2%)	190 (0.6%)	1.59 (1.10 to 2.29)
Valvular diseases	42 (1.4%)	189 (0.6%)	1.52 (1.07 to 2.16)
Gout	425 (14.1%)	2051 (6.8%)	1.48 (1.31 to 1.67)
Acute myocardial infarction	268 (8.9%)	1153 (3.8%)	1.48 (1.28 to 1.72)
Use of oral anticoagulant agents	565 (18.7%)	2802 (9.3%)	1.47 (1.32 to 1.64)
Other diseases of the nervous system	45 (1.5%)	259 (0.9%)	1.46 (1.05 to 2.03)
Use of corticosteroids	609 (20.1%)	4011 (13.3%)	1.43 (1.29 to 1.58)
Malignant neoplasms	238 (7.9%)	1460 (4.8%)	1.41 (1.21 to 1.63)
Other diseases of circulatory system	207 (6.8%)	1022 (3.4%)	1.41 (1.20 to 1.66)

Depression	676 (22.4%)	4234 (14.0%)	1.40 (1.27 to 1.55)
Chronic Obstructive Pulmonary Disease, asthma	510 (16.9%)	3320 (11.0%)	1.37 (1.23 to 1.52)
Arrhythmia	355 (11.7%)	1811 (6.0%)	1.33 (1.17 to 1.52)
Coronary and peripheral vascular diseases	521 (17.2%)	3391 (11.2%)	1.32 (1.19 to 1.47)
Diseases of the skin and of subcutaneous tissues	81 (2.7%)	498 (1.6%)	1.31 (1.02 to 1.67)
Epilepsy and recurrent seizures	133 (4.4%)	839 (2.8%)	1.29 (1.06 to 1.57)
Chronic pain	291 (9.6%)	1741 (5.8%)	1.27 (1.11 to 1.46)
Parkinson disease and parkinsonism	108 (3.6%)	591 (2.0%)	1.22 (1.02 to 1.57)

Footnote. Analysis included 3,023 patients who, starting from at least 14 days after completing scheduled vaccine, experienced severe COVID-119 illness documented by hospital admission inclusive in Intensive Care Unit, or death (severe cases), and 30,230 controls randomly selected to be 1:10 matched for date of vaccination completion and municipality of residence, and for not having yet experienced the infection on the date on which the corresponding case experienced it (index date). Among the 43 conditions tested (i.e., that portion of 59 candidate predictors of whom at least ten cases suffered), the 34 ones significantly associated with the considered outcome are listed in the Table. Adjusted conditional logistic regression models were fitted for estimating adjusted odds ratio, OR (point estimate of the association strength) and the corresponding 95% confidence interval (CI)
